# Unraveling the Clinical Features and Outcomes of IgG4-Related Ophthalmic Disease

**DOI:** 10.3390/jcm13133780

**Published:** 2024-06-27

**Authors:** Doah Kim, SangYoon Jeong, Helen Lew

**Affiliations:** 1 Department of Ophthalmology, Bundang CHA Medical Center, CHA University, Bundang-gu, Seongnam 13496, Gyeonggi-do, Republic of Korea; a216015@chamc.co.kr; 2 Department of Rheumatology, Bundang CHA Medical Center, CHA University, Bundang-gu, Seongnam 13496, Gyeonggi-do, Republic of Korea; jungsy7597@cha.ac.kr

**Keywords:** IgG4-related ophthalmic disease, corticosteroids, steroid-sparing agent, azathioprine, mycophenolate mofetil, hydroxychloroquine, clinical outcomes

## Abstract

**Background/Objectives**: IgG4-related ophthalmic disease (IgG4-ROD), characterized by lymphoplasmacytic infiltration, fibrosis, and elevated IgG4 levels, presents diagnostic challenges while offering insights into immune-mediated inflammatory disorders. The aim of this study was to comprehensively examine the clinical features and outcomes of IgG4-ROD. **Materials and Methods:** A retrospective study was conducted on 33 patients diagnosed with IgG4-ROD, fulfilling the American College of Rheumatology/European League Against Rheumatism (ACR/EULAR) criteria. The demographic characteristics of the IgG4-ROD patients were compared with those of 37 patients diagnosed with IgG4-related disease (IgG4-RD) in departments other than ophthalmology (IgG4-nonROD) at the same hospital during the same period. The patients diagnosed with IgG4-ROD were initially treated with glucocorticosteroid (GCS) monotherapy, GCS combined with steroid-sparing agents (SSAs; mycophenolate mofetil, azathioprine, hydroxychloroquine), biologic agent (rituximab) monotherapy, or watchful waiting. The primary outcome was the assessed treatment response at 6 months, and the secondary outcome was the evaluation of recurrence at 1 year after initial treatment. A response was evaluated as the absence of ocular signs and symptoms, either clinically or radiologically. **Results:** Eyelid swelling (17 patients, 51.5%) was the most common symptom, and lacrimal gland (17 patients, 51.5%) was the most frequent site of involvement. The response rate for GCS monotherapy was 33.3% (3 out of 9 patients), while the response rate for GCS combined with SSA was 60.0% (9 out of 15 patients). The lacrimal gland group demonstrated a significantly higher treatment response compared to the non-lacrimal gland group (66.7% vs. 20.0%, *p* = 0.013), and the combination of GCS and SSA resulted in a significantly higher treatment response than the GCS monotherapy (77.8% vs. 33.3%, *p* = 0.045). The group including hydroxychloroquine (HCQ), which comprised 5 out of 33 patients (15.2%), showed no recurrence at 1 year. **Conclusions:** The combination therapy of GCS and SSA for IgG4-ROD can be considered an effective treatment approach and HCQ could be considered as a potential adjunctive therapy for IgG4-ROD.

## 1. Introduction

IgG4-related ophthalmic disease (IgG4-ROD), characterized by lymphoplasmacytic infiltration, tissue fibrosis, and elevated serum IgG4 levels, has emerged as a rapidly growing area of interest in ophthalmology and rheumatology due to its diverse manifestations and complex underlying pathophysiology. Despite the diagnostic challenges, IgG4-ROD offers valuable insights into the mechanisms of immune-mediated inflammatory disorders [1].

Periorbital manifestations of IgG4-ROD include eyelid swelling (unilateral or bilateral), exophthalmos, ptosis (drooping of the eyelid), and conjunctival injection [2]. The swelling is often gradual in onset and can mimic other orbital inflammatory processes. Proptosis may occur due to the mass effect of intra-orbital inflammatory lesions. Compression of the extraocular muscles by these lesions can lead to diplopia, such as horizontal diplopia, and limitations in ocular motility. Systemic symptoms, such as fatigue, weight loss, and fever, may be present, particularly in patients with multi-organ involvement [3].

Despite its recent recognition as a distinct clinicopathologic entity, IgG4-ROD has garnered significant global attention due to its diverse clinical presentations and potential for significant morbidity if left untreated. IgG4-ROD exhibits a predilection for certain populations, with studies demonstrating significant disparities in prevalence between East Asian and Western cohorts [4,5]. This geographic variability emphasizes the critical need to elucidate the diverse epidemiological and clinical characteristics of IgG4-ROD across distinct populations.

The treatment options for IgG4-ROD have undergone a rapid expansion in recent years, with the spectrum of modalities including systemic glucocorticosteroid (GCS), novel biologic agents, and surgical intervention [6,7]. Although these advancements have yielded improved outcomes and quality of life for IgG4-ROD patients, uncertainties remain regarding the optimal treatment selection, timing, and long-term management [7].

We investigated the clinical characteristics and outcomes of IgG4-ROD patients at our hospital, focusing on specific disease manifestations. We comprehensively analyzed the demographic data, presenting symptoms, diagnostic pathways, treatment modalities used, and long-term outcomes. The results of the analysis will contribute to the development of more personalized and effective management strategies for IgG4-ROD patients.

We elucidated the complexities of IgG4-ROD through meticulous examination of the clinical data and rigorous statistical analysis. Our findings have the potential to directly inform clinical practice and guide therapeutic decision-making, ultimately improving patient management for this complex ophthalmic condition. This comprehensive understanding of IgG4-ROD may lead to significant optimization of patient care and outcomes.

## 2. Patients and Methods

We retrospectively analyzed the medical records of 33 patients with IgG4-ROD who presented at Bundang CHA Hospital between July 2016 and September 2023; all the patients had a follow-up exceeding 6 months. During the same period, we also analyzed the records of 37 patients diagnosed with IgG4-related disease (IgG4-RD) in departments other than ophthalmology (IgG4-nonROD) at Bundang CHA Hospital. Thus, a total of 70 patient records were collected. We compared the clinical characteristics of IgG4-ROD and IgG4-nonROD by analyzing the demographic data of the two groups. All the patients fulfilled the 2019 American College of Rheumatology/European League Against Rheumatism criteria for IgG4-related disease [8,9]. Of the 33 IgG4-ROD patients, 31 presented to the ophthalmology department, whereas the 2 remaining patients were referred from other departments due to ocular complaints. Clinical records were reviewed to extract data on the demographic characteristics, clinical features, treatment modalities, and patient outcomes. Patients with pre-existing ophthalmic or autoimmune rheumatic diseases, active or severe infections, active cancer, or conditions that could mimic IgG4-RD were excluded.

The patients were initially treated with GCS monotherapy (30 mg oral prednisolone tapered by 5 mg every 1–2 weeks based on disease activity), GCS combined with steroid-sparing agents (SSAs), the biologic agent rituximab as monotherapy, or watchful waiting. Patients who did not respond to GCS were subsequently initiated on combination therapy with an SSA, such as mycophenolate mofetil (MMF), azathioprine (AZA), or hydroxychloroquine (HCQ).

The primary outcome evaluated the treatment success at 6 months post-treatment. Response was defined as complete clinical and radiological resolution of ocular signs and symptoms. No response indicated no improvement or disease worsening. Relapse, defined as the recurrence of ocular lesions clinically and radiologically during GCS tapering, was assessed based on the established criteria [10,11,12]. The secondary outcome, evaluated at 1 year post-treatment, assessed recurrence, defined as the reappearance of lesions in the ocular adnexa after treatment discontinuation [13].

The Institutional Review Board of Bundang CHA Hospital (IRB File No. CHAMC 2024-01-005-002, Approval date: 19 January 2024) approved the study protocol.

The statistical analyses were performed using SPSS (version 29.0; IBM Corporation, Armonk, NY, USA). Numerical data are presented as the mean ± standard deviation for the normally distributed variables. For comparing the categorical variables, the chi-square test or Fisher’s exact test was employed, and for the continuous variables, the independent 2-sample *t*-test was used. A *p*-value < 0.05 was considered statistically significant.

## 3. Results

### 3.1. Baseline Characteristics of Patients with IgG4-nonROD and IgG4-ROD

**Figure 1:**
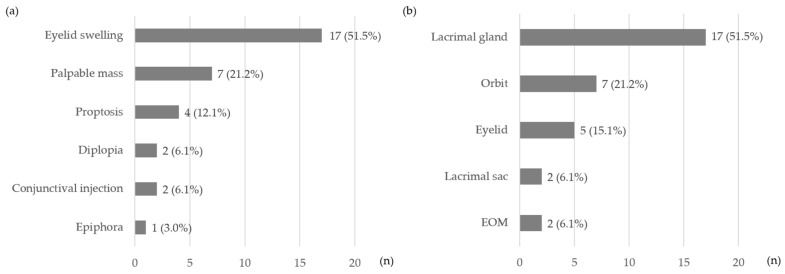
Among patients presenting to the ophthalmology department, the number of patients by (**a**) symptom and (**b**) affected site. EOM, extraocular muscle.

**Figure 2:**
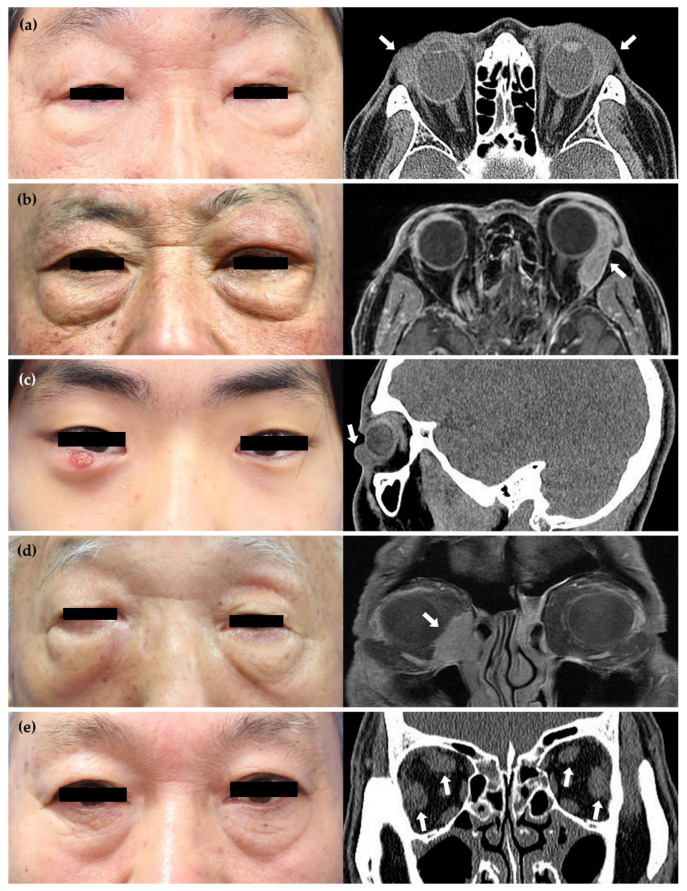
Gross photo (**left**) and radiological image (**right**) according to the 5 affected sites (white arrow) of IgG4-ROD. (**a**) Lacrimal gland: a 71-year-old male presented with bilateral eyelid swelling, and a facial CT showed enlargements of the bilateral lacrimal glands, more pronounced on the left. (**b**) Orbit: a 73-year-old male presented with swelling of the left eyelid, and an orbit MRI with CM showed a 3.3 × 1.2 × 1.9 cm lobulated enhancing mass in the superolateral aspect of the extraconal space in the left orbit. (**c**) Eyelid: a 20-year-old male presented with a mass in the right eyelid, and a facial CT revealed about 0.5 × 0.8 × 1.1 cm of soft tissue-attenuated swelling in the right lower eyelid. (**d**) Lacrimal sac: a 76-year-old male presented with swelling of the right eyelid, and an orbit MRI with CM showed a 3.0 × 2.5 cm lobulated soft tissue mass in the right anteroinferomedial orbit. (**e**) EOM: a 60-year-old male complained of diplopia, and a facial CT showed severe diffuse enlargements of the bilateral superior and lateral rectus muscles. IgG4-ROD, IgG4-related ophthalmic disease; CT, computed tomography; MRI; magnetic resonance imaging; CM, contrast media; EOM, extraocular muscle.

In total, 70 patients were included, comprising 37 IgG4-nonROD and 33 IgG4-ROD patients. The mean age of the IgG4-ROD patients was significantly younger than the IgG4-nonROD patients (*p* = 0.006). There were no significant differences between the IgG4-nonROD and IgG4-ROD groups in terms of sex, serum IgG4 level, elevated pre-treatment serum IgG4 level, follow-up duration, and the proportion meeting the diagnostic criteria (Table 1). The initial departments visited by the total 70 patients were as follows: ophthalmology with 31 patients (44.3%), otolaryngology (ENT) with 12 patients (17.2%), gastroenterology (GI) with 8 patients (11.4%), pulmonology (PLM) with 8 patients (11.4%), rheumatology (RHE) with 4 patients (5.7%), urology (URO) with 3 patients (4.3%), general surgery (GS) with 2 patients (2.9%), cardiology (CV) with 1 patient (1.4%), and nephrology (NPH) with 1 patient (1.4%). Among the patients who visited the ophthalmology department, the symptoms were as follows: eyelid swelling was the most common (51.5%), followed by palpable mass (21.2%), proptosis (12.1%), diplopia (6.1%), conjunctival injection (6.1%), and epiphora (3.0%). The lacrimal gland (51.5%) was the most frequently affected site, followed by the orbit (21.2%), eyelid (15.1%), lacrimal sac (6.1%), and extraocular muscles (EOM) (6.1%) (Figure 1 and Figure 2).

### 3.2. Treatment and Outcomes of Patients with IgG4-ROD

**Figure 3:**
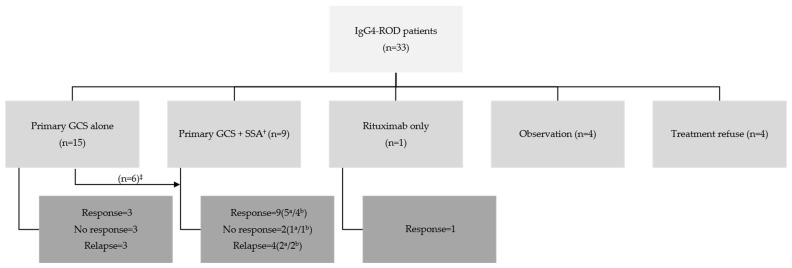
Treatment responses in the IgG4-ROD patients at 6 months after initial treatment. IgG4-ROD, IgG4-related ophthalmic disease; GCS, glucocorticosteroid; SSA, steroid-sparing agent. The SSAs included mycophenolate mofetil, azathioprine, and hydroxychloroquine. ^†^ SSA was divided into two groups: one comprised only mycophenolate mofetil (*n* = 3) or azathioprine (*n* = 1), and the other comprised mycophenolate mofetil plus hydroxychloroquine (*n* = 5). ^‡^ The initial treatment was GCS alone, and a group received additional SSA therapy during treatment. ^a^ Only mycophenolate mofetil or azathioprine group. ^b^ Mycophenolate mofetil plus hydroxychloroquine group.

In total, 33 IgG4-ROD patients received various initial treatment regimens. These regimens included GCS monotherapy (*n* = 15), combination therapy with GCS and SSA (*n* = 9), rituximab (*n* = 1), observation (*n* = 4), and treatment refusal (*n* = 4) (Figure 3). In the GCS monotherapy group, considering the treatment response and serum IgG4 levels during follow-up, 6 patients were additionally administered SSAs. Of the 9 patients treated with GCS alone, 3 patients (33.3%) experienced a response, 3 patients (33.3%) exhibited no response, and 3 patients (33.3%) relapsed at 6 months. In the combination therapy group, the specific SSAs used with corticosteroids included MMF, AZA, and a combination of MMF and HCQ in three, one, and five patients, respectively. Including the six patients who started on GCS alone and later added an SSA, out of a total of fifteen patients, nine patients (60.0%) showed a response, two (13.3%) had no response, and four (26.7%) relapsed. Notably, a patient receiving rituximab responded at the 6-month follow-up. In the observation group, four patients (12.1%) were monitored without receiving any treatment, two patients did not need treatment after biopsy, while the other two patients were unable to take GCS due to their underlying medical conditions: one had diabetes and the other had liver cirrhosis. Four patients (12.1%) refused treatment against medical advice.

Among the 33 IgG4-ROD patients, the mean age of the no-response group (5 patients) to initial treatment was 30.40 ± 22.40 years, which was statistically significantly lower than that of the response group (20 patients), with a mean age of 54.40 ± 13.84 years (*p* = 0.006). No significant differences were observed in the other clinical characteristics (Table 2).

**Figure 4:**
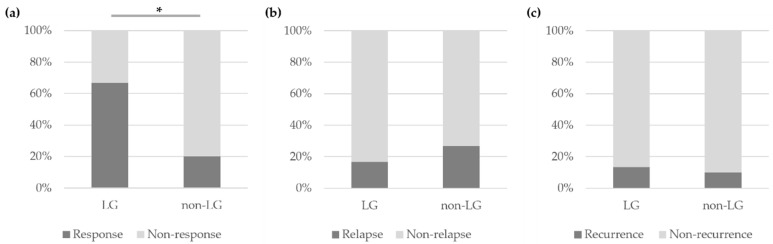
According to the involvement site in IgG4-ROD, comparison of the (**a**) response rates and (**b**) relapse rates at 6 months after treatment, and the (**c**) recurrence rates at 1 year after treatment between the lacrimal gland involvement and non-lacrimal gland involvement groups. LG, lacrimal gland. * *p*-value = 0.013.

At 6 months after the initial treatment, the lacrimal gland involvement group experienced a significantly higher response rate than the non-lacrimal gland involvement group (66.7% vs. 20.0%, *p* = 0.013). There was no statistically significant difference in the relapse rates at 6 months and the recurrence rates at 1 year after initial treatment between the lacrimal involvement and non-lacrimal involvement groups (Figure 4).

**Figure 5:**
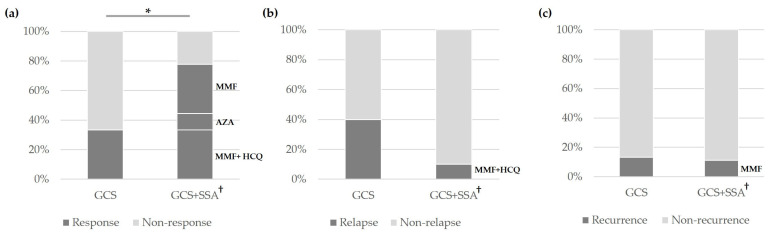
According to the initial treatment, comparison of the (**a**) response rates and (**b**) relapse rates at 6 months after treatment and the (**c**) recurrence rates at 1 year after treatment. GCS, corticosteroid; SSA, steroid-sparing agent; MMF, mycophenolate mofetil; AZA, azathioprine; HCQ, hydroxychloroquine. * *p*-value = 0.045 2020 GCS + SSA consists of 3 methods: GCS + MMF, GCS + AZA, and the GCS + MMF + HCQ.

Combination therapy with GCS and SSA resulted in a significantly higher response rate compared to GCS monotherapy (77.8% vs. 33.3%, *p* = 0.045). The response for the administration of GCS and SSA in the combination groups consisted of 33.3% combination with GCS and MMF, 11.1% combination with GCS and AZA, and 33.3% combination with GCS, MMF, and HCQ. In the GCS and SSA combination therapy group, relapses occurred in the GCS, MMF, and HCQ combination subgroup, and recurrences were seen in the GCS and MMF combination subgroup. There was no significant difference in the relapse rate at 6 months and the recurrence rate at 1 year after treatment when comparing GCS monotherapy to combination therapy with GCS and SSA (Figure 5).

## 4. Discussion

We investigated the treatment responses and long-term outcomes in patients with IgG4-ROD. Consistent with prior studies, GCS was the first-line therapy for most patients (72.7%, *n* = 24). 

In our study, 5 out of 33 IgG4-ROD patients (15.2%) showed no response, including 3 patients on GCS monotherapy and 2 patients on a combination of GCS and SSA therapy. Son et al. [14] reported that two patients (6.7%) who received steroid monotherapy showed no response. In a large cohort study in China, the rates of maintaining long-term corticosteroid therapy were 12% (9/75) in those receiving primary corticosteroid alone and 28.6% (2/7) in those receiving primary corticosteroid in combination with SSA/biologics [6]. Limited literature exists regarding the risk factors for no response in IgG4-ROD, and there is insufficient evidence to define the factors associated with no response. However, our study revealed that the mean age of the no-response group was significantly lower than that of the response group (30.4 ± 22.4 vs. 54.4 ± 13.8 years, *p* = 0.006). This result demonstrates a similar trend to the risk factors for relapse. In a Japanese study, male gender and younger age at disease onset were identified as risk factors for relapse, possibly due to the interaction between sex hormones and the immune response [15]. In a French study, all the patients who received prednisone as the initial treatment showed clinical improvement, but approximately two-thirds of them experienced a recurrence, indicating a high relapse rate [16]. In all the cases of recurrence, sclerosing fibrotic changes were present; however, it remains unclear whether the lesions progressively sclerose over time or if this represents the primary sclerosing process [17].

The multifaceted nature of IgG4-RD manifests in various organs, including the salivary glands, orbits, retroperitoneum, prostate, and others [18,19,20,21], consistent with our findings. In our study, 70 patients received care across nine different specialties, emphasizing the complex and systemic nature of IgG4-RD. The lacrimal gland is the most frequently involved orbital structure, with reported involvement ranging from 62 to 88% [2,22,23]. Consistent with these findings, we identified the lacrimal gland as the most commonly affected site (51.5%, *n* = 17). Yuka et al. [24] reported involvement in areas other than the lacrimal gland in 52.3% of cases, and Lee et al. [25] described a case of IgG4-ROD involving the caruncle, emphasizing the complex nature of IgG4-ROD. 

Disparities in the IgG4-ROD prevalence between East Asians and Western populations arise from a complex interplay of factors. Although genetic predisposition is a potential contributor, environmental factors, such as dietary habits and pathogen exposure, might also play a role. Furthermore, variations in diagnostic awareness and practices across regions could further influence the reported IgG4-ROD prevalence [4,5].

Orbital magnetic resonance imaging (MRI) or computed tomography (CT) can demonstrate the characteristic findings of IgG4-ROD, including diffuse orbital infiltration, lacrimal gland enlargement, and thickening of orbital soft tissues [26]. However, definitive diagnosis of IgG4-ROD relies on the histopathological findings of biopsy specimens from the affected orbital tissues. The key features on histology include dense lymphoplasmacytic infiltrates, storiform fibrosis, and obliterative phlebitis, accompanied by an increased number of IgG4-positive plasma cells [27]. Although elevated serum IgG4 levels (>135 mg/dL) are frequently observed in IgG4-RD, they lack specificity for IgG4-ROD. Therefore, diagnosis requires a comprehensive evaluation integrating clinical presentation, radiological findings, and histopathological features [28]. The variable pathological presentation across organs necessitates a multidisciplinary approach, with clinicians and pathologists working together to integrate clinical manifestations, radiological findings, and histopathological features for accurate diagnosis and optimal patient management. 

Systemic GCS, including prednisone or prednisolone, is the mainstay of the first-line therapy for IgG4-ROD. Initial treatment typically involves a corticosteroid dosage of 30–40 mg/day, followed by a gradual tapering to maintain remission [11,12,29]. Wu et al. [12] reported no significant difference in the remission induction rates between the high- and medium-dose glucocorticoid groups. Among the 10 patients who met the IgG4-RD classification criteria, 2 out of 3 patients (66.7%) who received initial treatment with radiation therapy did not respond well to the treatment. Corticosteroid therapy improved the clinical signs and symptoms in most patients, but two out of six patients (33.3%) experienced a relapse [30]. Detiger et al. [11] demonstrated a response rate of 89%, compared to our observed rate of 33.3%. These discrepancies may be attributed to variations in the study populations, response definitions, and follow-up durations. For GCS-resistant or relapsing disease, SSAs, such as AZA, MMF, or rituximab, can be considered [6,31,32]. Surgical debulking or orbital decompression may be necessary in severe cases of orbital congestion, compressive optic neuropathy, or refractory disease [33]. However, surgery typically remains reserved for patients who fail to respond adequately to medical management [34].

Rituximab, an anti-CD20 monoclonal antibody, has become a mainstay in the management of IgG4-RD, demonstrating efficacy in remission induction and management of disease flares [35,36,37]. Several studies have demonstrated that rituximab can be the sole agent for remission induction [36], whereas others emphasize its potential to prevent relapses [38]. However, the treatment response and duration remain variable, with approximately 40% of patients achieving remission at 1 year after rituximab induction [34]. Although rituximab effectively induces remission in most IgG4-RD patients, the optimal long-term management strategy remains uncertain [18]. Hydroxychloroquine, initially developed as an antimalarial drug, has gained wider use in rheumatic and autoimmune diseases due to its anti-inflammatory and potential anti-fibrotic properties [39,40,41]. Karim et al. [39] reported that hydroxychloroquine used as maintenance therapy achieved a complete response in 50% of patients. Notably, 20.8% of patients received HCQ as the initial therapy. Although the treatment response rates did not differ significantly between the groups, no relapses were observed in the HCQ-treated group, and no recurrences were identified in this group. Although larger studies are needed to evaluate HCQ’s efficacy, it may be a promising second- or third-line therapy for less severe IgG4-RD, reducing relapse rates and facilitating glucocorticoid discontinuation [39,42].

Our study had several limitations. First, its retrospective design necessitates a cautious interpretation of the findings. Second, it was conducted at a single institution with a relatively small number of participants, warranting multicenter studies with a larger sample size for broader generalization. Third, the outcomes were categorized based on the initial treatment modality.

**Figure 6:**
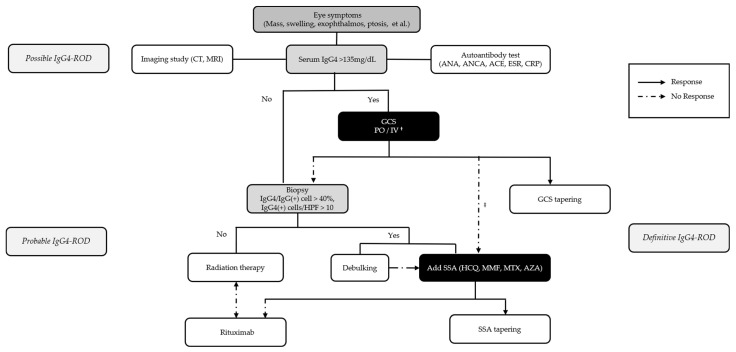
Flow chart of the management of patients with suspected IgG4-related ophthalmic disease based on clinical and historical data. GCS, glucocorticosteroid; PO, per oral; IV, intravenous; SSA, steroid-sparing agent; HCQ, hydroxychloroquine; MMF, mycophenolate mofetil; MTX, methotrexate; AZA, azathioprine. ^†^: For severe inflammation or vision-threatening conditions, intravenous steroid therapy may be administered. ^‡^: Less likelihood of tumorous lesions or refusal of biopsy.

Based on our findings, we propose a treatment flow chart (Figure 6) to guide the management of patients with IgG4-ROD. This chart integrates the initial treatment strategies and management plans based on the treatment response, highlighting the importance of individualized patient care.

## 5. Conclusions

In conclusion, our analysis of IgG4-ROD patients revealed diverse clinical presentations, necessitating consultations across various medical specialties. The lacrimal gland was the most frequently affected site; patients with this presentation demonstrated a superior response to treatment compared to those without. Although the initial management of IgG4-ROD primarily relied on GCS monotherapy, considering immunopathophysiology based on the clinical outcomes of combined steroid and immunosuppressive therapy, a combination of GCS and SSA therapy may represent a more effective treatment approach. Furthermore, HCQ could be considered as a potential adjunctive therapy for IgG4-ROD.

## Figures and Tables

**Table 1:** Clinical characteristics of patients with IgG4-nonROD and IgG4-ROD at baseline.

	IgG4-nonROD (*n* = 37)	IgG4-ROD (*n* = 33)	*p*-Value
Age, years ± SD	59.24 ± 14.65	47.52 ± 19.50	0.006 *
Male, *n* (%)	26 (70.27)	19 (57.58)	0.27 ^†^
Serum IgG4 level, mg/dL ± SD	215.81 ± 97.60	292.37 ± 250.22	0.79 *
Elevated pre-treatment serum IgG4 level, *n* (%)	31 (83.78)	28 (84.85)	1.00 ^‡^
Follow-up duration, months ± SD	11.49 ± 6.61	16.64 ± 11.79	0.06 *
Diagnostic criteria			
Definitive ^a^	8 (21.62)	9 (27.27)	0.78 ^‡^
Probable ^b^	6 (16.22)	5 (15.15)	1.00 ^‡^
Possible ^c^	23 (62.16)	19 (57.58)	0.81 ^‡^

Bold values are significant. * *t*-test. ^†^ chi-square test. ^‡^ Fisher’s exact test. IgG4-nonROD, IgG4-non related ophthalmic disease; IgG4-ROD, IgG4-related ophthalmic disease; SD, standard deviation; *n*, number. ^a^ = combination of 1 + 2 + 3; ^b^ = combination of 1 + 3; ^c^ = combination of 1 + 2; 1. Diffuse or localized swelling or masses in single or multiple organs; 2. Serum IgG4 levels ≥ 135 mg/dl; 3. Histology (1) lymphoplasmacytic infiltration and fibrosis, (2) IgG4-positive plasma cells: ratio of IgG4/IgG positive cells >40%, and <10 IgG4-positive plasma cells/high power field.

**Table 2:** Comparison of the clinical characteristics between the no-response and response groups in the IgG4-ROD patients.

	No Response (*n* = 5)	Response (*n* = 20)	*p*-Value
Age, years ± SD	30.40 ± 22.40	54.40 ± 13.84	0.006 *
Male, *n* (%)	3 (60.0)	13 (65.0)	1.00 ^†^
Serum IgG4 level, mg/dL ± SD	137.46 ± 56.88	333.23 ± 305.06	0.53 *
Elevated pre-treatment serum IgG4 level, *n* (%)	5 (100.00)	17 (85.00)	1.00 ^‡^
Follow-up duration, months ± SD	19.43 ± 7.77	18.23 ± 13.89	0.41 *
Diagnostic criteria, *n* (%)			
Definitive ^a^	1 (20.00)	8 (40.00)	0.62 ^‡^
Probable ^b^	0 (0.00)	1 (5.00)	1.00 ^‡^
Possible ^c^	4 (80.00)	11 (55.00)	0.62 ^‡^

Bold values are significant. * *t*-test. ^†^ chi-square test. ^‡^ Fisher’s exact test. IgG4-ROD, IgG4-related ophthalmic disease; SD, standard deviation; *n*, number. ^a^ = combination of 1 + 2 + 3; ^b^ = combination of 1 + 3; ^c^ = combination of 1 + 2; 1. Diffuse or localized swelling or masses in single or multiple organs; 2. Serum IgG4 levels ≥ 135 mg/dl; 3. Histology (1) lymphoplasmacytic infiltration and fibrosis, (2) IgG4-positive plasma cells: ratio of IgG4/IgG positive cells >40%, and <10 IgG4-positive plasma cells/high power field.

## Data Availability

The datasets analyzed during the current study are available from the corresponding author on reasonable requests.

## References

[1] Umehara H., Okazaki K., Kawa S., Takahashi H., Goto H., Matsui S., Ishizaka N., Akamizu T., Sato Y., Kawano M. (2021). The 2020 revised comprehensive diagnostic (RCD) criteria for IgG4-RD. Mod. Rheumatol..

[2] Wallace Z.S., Deshpande V., Stone J.H. (2014). Ophthalmic manifestations of IgG4-related disease: Single-center experience and literature review. Semin. Arthritis Rheum..

[3] Perugino C.A., Stone J.H. (2020). IgG4-related disease: An update on pathophysiology and implications for clinical care. Nat. Rev. Rheumatol..

[4] Arai H., Hayashi H., Takahashi K., Koide S., Sato W., Hasegawa M., Yamaguchi Y., Aten J., Ito Y., Yuzawa Y. (2015). Tubulointerstitial fibrosis in patients with IgG4-related kidney disease: Pathological findings on repeat renal biopsy. Rheumatol. Int..

[5] Cho S.H., Song T.J., Park J.S., Yoon J.H., Yang M.J., Yoon S.B., Lee J.M., Lee Y.N., Kim S.H., Choi E.K. (2023). Comparison of the long-term outcomes between proximal and distal IgG4-related sclerosing cholangitis: A multicenter cohort study. J. Gastroenterol. Hepatol..

[6] Lai K.K.H., Li E.Y.M., Chan R.Y.C., Chu W.C.W., Cheng A.C.O., Chan K.K.W., Chin J.K.Y., Kwok J.S.W., Io I.Y.F., Yip N.K.F. (2023). Treatment outcomes and their determinants of IgG4-related ophthalmic disease: A territory-wide cohort study. Br. J. Ophthalmol..

[7] Cai S., Hu Z., Chen Y., Zhong J., Dong L. (2022). Potential roles of non-lymphocytic cells in the pathogenesis of IgG4-related disease. Front. Immunol..

[8] Wallace Z.S., Zhang Y., Perugino C.A., Naden R., Choi H.K., Stone J.H., ACR/EULAR IgG4-RD Classification Criteria Committee (2019). Clinical phenotypes of IgG4-related disease: An analysis of two international cross-sectional cohorts. Ann. Rheum. Dis..

[9] Wallace Z.S., Naden R.P., Chari S., Choi H.K., Della-Torre E., Dicaire J.F., Hart P.A., Inoue D., Kawano M., Khosroshahi A. (2020). The 2019 American College of Rheumatology/European League Against Rheumatism classification criteria for IgG4-related disease. Ann. Rheum. Dis..

[10] Chen J., Zhang P., Ye H., Xiao W., Chen R., Mao Y., Ai S., Liu Z., Tang L., Yang H. (2021). Clinical features and outcomes of IgG4-related idiopathic orbital inflammatory disease: From a large southern China-based cohort. Eye.

[11] Detiger S.E., Karim A.F., Verdijk R.M., van Hagen P.M., van Laar J.A.M., Paridaens D. (2019). The treatment outcomes in IgG4-related orbital disease: A systematic review of the literature. Acta Ophthalmol..

[12] Wu Q., Chang J., Chen H., Chen Y., Yang H., Fei Y., Zhang P., Zeng X., Zhang F., Zhang W. (2017). Efficacy between high and medium doses of glucocorticoid therapy in remission induction of IgG4-related diseases: A preliminary randomized controlled trial. Int. J. Rheum. Dis..

[13] Masaki Y., Matsui S., Saeki T., Tsuboi H., Hirata S., Izumi Y., Miyashita T., Fujikawa K., Dobashi H., Susaki K. (2017). A multicenter phase II prospective clinical trial of glucocorticoid for patients with untreated IgG4-related disease. Mod. Rheumatol..

[14] Son K.Y., Woo K.I., Kim Y.D. (2022). Clinical Outcomes of IgG4-Related Ophthalmic Disease and Idiopathic Sclerosing Orbital Inflammation. Ophthalmic Plast. Reconstr. Surg..

[15] Yamamoto M., Nojima M., Takahashi H., Yokoyama Y., Ishigami K., Yajima H., Shimizu Y., Tabeya T., Matsui M., Suzuki C. (2015). Identification of relapse predictors in IgG4-related disease using multivariate analysis of clinical data at the first visit and initial treatment. Rheumatology.

[16] Ebbo M., Patient M., Grados A., Groh M., Desblaches J., Hachulla E., Saadoun D., Audia S., Rigolet A., Terrier B. (2017). Ophthalmic manifestations in IgG4-related disease: Clinical presentation and response to treatment in a French case-series. Medicine.

[17] Andrew N., Sladden N., Kearney D., Crompton J., Selva D. (2014). Sequential biopsies from immunoglobulin G4-related orbital disease demonstrate progressive fibrosis. Clin. Exp. Ophthalmol..

[18] Katz G., Stone J.H. (2022). Clinical Perspectives on IgG4-Related Disease and Its Classification. Annu. Rev. Med..

[19] Sanchez-Oro R., Alonso-Munoz E.M., Marti Romero L. (2019). Review of IgG4-related disease. Gastroenterol. Hepatol..

[20] Maritati F., Peyronel F., Vaglio A. (2020). IgG4-related disease: A clinical perspective. Rheumatology.

[21] Adam Z., Zeman D., Cermak A., Dastych M., Doubkova M., Horvath T., Skorkovska S., Adamova Z., Rehak Z., Koukalova R. (2022). IgG4-related disease. Clinical manifestation differential diagnosis and recent International Diagnostic Criteria for IgG4-related disease. Vnitr. Lek..

[22] Martín-Nares E., Hernández-Molina G., Lima G., Hernández-Ramírez D.F., Chan-Campos I., Saavedra-González V., Llorente L. (2023). Tear levels of IL-7, IL-1α, and IL-1β may differentiate between IgG4-related disease and Sjögren’s syndrome. Clin. Rheumatol..

[23] Sato Y., Ohshima K., Ichimura K., Sato M., Yamadori I., Tanaka T., Takata K., Morito T., Kondo E., Yoshino T. (2008). Ocular adnexal IgG4-related disease has uniform clinicopathology. Pathol. Int..

[24] Sogabe Y., Ohshima K., Azumi A., Takahira M., Kase S., Tsuji H., Yoshikawa H., Nakamura T. (2014). Location and frequency of lesions in patients with IgG4-related ophthalmic diseases. Graefes Arch. Clin. Exp. Ophthalmol..

[25] Lee G.M., Kim N., Paik J.H. (2022). Immunoglobulin G4-related Ophthalmic Disease of the Caruncle: A Case Report. Korean J. Ophthalmol..

[26] Yoo R.E., Park S.W., Rhim J.H., Kim J.E., Kim S.C., Choe J.Y., Choung H.K., Khwarg S.I., Kim J.H., Lee J.H. (2020). CT and MR imaging findings of ocular adnexal mucosa-associated lymphoid tissue lymphoma associated with IgG4-related disease: Multi-institutional case series. Int. J. Ophthalmol..

[27] Yuan Y., Meng F., Ren H., Yue H., Xue K., Zhang R. (2022). Pathological count of IgG4-positive plasmacytes suggests extraophthalmic involvement and relapse in patients with IgG4-related ophthalmic disease: A retrospective study. Arthritis Res. Ther..

[28] Choi S.J., Ahn S.M., Oh J.S., Hong S., Lee C.K., Yoo B., Kim Y.G. (2023). Serum IgG4 level during initial treatment as a predictor of relapse in IgG4-related disease. PLoS ONE.

[29] Kamisawa T., Okazaki K., Kawa S., Ito T., Inui K., Irie H., Nishino T., Notohara K., Nishimori I., Tanaka S. (2014). Amendment of the Japanese Consensus Guidelines for Autoimmune Pancreatitis, 2013 III. Treatment and prognosis of autoimmune pancreatitis. J. Gastroenterol..

[30] Kubota T., Moritani S., Katayama M., Terasaki H. (2010). Ocular adnexal IgG4-related lymphoplasmacytic infiltrative disorder. Arch. Ophthalmol..

[31] Peng Y., Li J.Q., Zhang P.P., Zhang X., Peng L.Y., Chen H., Zhou J.X., Zhang S.Z., Yang H.X., Liu J.J. (2019). Clinical outcomes and predictive relapse factors of IgG4-related disease following treatment: A long-term cohort study. J. Intern. Med..

[32] Zhao Z., Mou D., Wang Z., Zeng Q., Wang Z., Xue J., Ren L., Liu Y., Su Y. (2021). Clinical features and relapse risks of IgG4-related ophthalmic disease: A single-center experience in China. Arthritis Res. Ther..

[33] Gupta N., Mathew J., Mohan H., Chowdhury S.D., Kurien R.T., Christopher D.J., Thangakunam B., Alexander M., Sivadasan A., Tamilarasi V. (2018). Addition of second-line steroid sparing immunosuppressants like mycophenolate mofetil improves outcome of Immunoglobulin G4-related disease (IgG4-RD): A series from a tertiary care teaching hospital in South India. Rheumatol. Int..

[34] Ominato J., Oyama T., Cho H., Shiozaki N., Umezu H., Takizawa J., Fukuchi T. (2019). The natural course of IgG4-related ophthalmic disease after debulking surgery: A single-centre retrospective study. BMJ Open Ophthalmol..

[35] Carruthers M.N., Topazian M.D., Khosroshahi A., Witzig T.E., Wallace Z.S., Hart P.A., Deshpande V., Smyrk T.C., Chari S., Stone J.H. (2015). Rituximab for IgG4-related disease: A prospective, open-label trial. Ann. Rheum. Dis..

[36] Ebbo M., Grados A., Samson M., Groh M., Loundou A., Rigolet A., Terrier B., Guillaud C., Carra-Dalliere C., Renou F. (2017). Long-term efficacy and safety of rituximab in IgG4-related disease: Data from a French nationwide study of thirty-three patients. PLoS ONE.

[37] Khosroshahi A., Carruthers M.N., Deshpande V., Unizony S., Bloch D.B., Stone J.H. (2012). Rituximab for the treatment of IgG4-related disease: Lessons from 10 consecutive patients. Medicine.

[38] Campochiaro C., Della-Torre E., Lanzillotta M., Bozzolo E., Baldissera E., Milani R., Arcidiacono P.G., Crippa S., Falconi M., Dagna L. (2020). Long-term efficacy of maintenance therapy with Rituximab for IgG4-related disease. Eur. J. Intern. Med..

[39] Karim A.F., Bansie R.D., Rombach S.M., Paridaens D., Verdijk R.M., van Hagen P.M., Van Laar J.A.M. (2018). The treatment outcomes in IgG4-related disease. Neth. J. Med..

[40] Muller-Calleja N., Manukyan D., Canisius A., Strand D., Lackner K.J. (2017). Hydroxychloroquine inhibits proinflammatory signalling pathways by targeting endosomal NADPH oxidase. Ann. Rheum. Dis..

[41] Browning D.J. (2002). Hydroxychloroquine and chloroquine retinopathy: Screening for drug toxicity. Am. J. Ophthalmol..

[42] Gan L., Luo X., Fei Y., Peng L., Zhou J., Li J., Lu H., Liu Z., Zhang P., Liu X. (2021). Long-Term Outcomes of IgG4-Related Ophthalmic Disease in a Chinese IgG4-Related Disease Cohort. Front. Med..

